# Ricci Curvature-Based Semi-Supervised Learning on an Attributed Network

**DOI:** 10.3390/e23030292

**Published:** 2021-02-27

**Authors:** Wei Wu, Guangmin Hu, Fucai Yu

**Affiliations:** School of Information and Communication Engineering, University of Electronic Science and Technology of China, Chengdu 611731, China; weiwucz@163.com (W.W.); hgm@uestc.edu.cn (G.H.)

**Keywords:** attributed network, cross entropy, Ricci curvature

## Abstract

In recent years, on the basis of drawing lessons from traditional neural network models, people have been paying more and more attention to the design of neural network architectures for processing graph structure data, which are called graph neural networks (GNN). GCN, namely, graph convolution networks, are neural network models in GNN. GCN extends the convolution operation from traditional data (such as images) to graph data, and it is essentially a feature extractor, which aggregates the features of neighborhood nodes into those of target nodes. In the process of aggregating features, GCN uses the Laplacian matrix to assign different importance to the nodes in the neighborhood of the target nodes. Since graph-structured data are inherently non-Euclidean, we seek to use a non-Euclidean mathematical tool, namely, Riemannian geometry, to analyze graphs (networks). In this paper, we present a novel model for semi-supervised learning called the Ricci curvature-based graph convolutional neural network, i.e., RCGCN. The aggregation pattern of RCGCN is inspired by that of GCN. We regard the network as a discrete manifold, and then use Ricci curvature to assign different importance to the nodes within the neighborhood of the target nodes. Ricci curvature is related to the optimal transport distance, which can well reflect the geometric structure of the underlying space of the network. The node importance given by Ricci curvature can better reflect the relationships between the target node and the nodes in the neighborhood. The proposed model scales linearly with the number of edges in the network. Experiments demonstrated that RCGCN achieves a significant performance gain over baseline methods on benchmark datasets.

## 1. Introduction

Many real-world systems can be modeled as networks, such as technological networks, social networks, citation networks and biological networks. The conventional approach, which uses a set of edges to represent a network, has proven that it is difficult to process complicated network data.

In recent years, a novel approach called network embedding has been attracting more and more attention. Network embedding, as an efficient tool for data mining, is designed to convert the information within the network to a continuous low-dimensional vector representation. The resulting representation sets the stage for many applications, including node classification [1], link prediction [2], community detection and visualization.

Network embedding approaches can be roughly divided into three categories: random walk-based approaches, matrix factorization-based algorithms and deep learning-based methods. Inspired by word embedding techniques in natural language processing [3], Deepwalk [4] trains the representation of each node in the network using the skip-gram model, treating sequences of nodes from truncated random walks as sentences and nodes as words. To use the skip-gram model, Deepwalk sets up a window to retrieve the context of the target word from the sequences of nodes, which is essentially the generalized neighborhood of the node. Node2vec [5] uses a random walk strategy different from the one in Deepwalk, called biased second-order random walking. By tuning the super parameters p and q, Node2vec can combine two sampling methods, which are called BFS and DFS, so that the neighborhood obtained by sampling can take into account both homogeneity and structural equivalence. LINE [6] takes the set of neighbors within two hops of the target node as its neighborhood, and it obtains the embedding of a network by preserving first-order and second-order proximities. The methods mentioned above, including Deepwalk, Node2vec and LINE, only consider the proximity of nodes in the network topology, but do not consider the additional information of nodes. FANE [7] generates a new network by adding virtual attribute nodes and virtual edges, and modifies the random walk strategy in Node2vec, so that the random walk can pass through the virtual attribute nodes. In this way, topological structure information and attribute information are integrated into the learning process of network embedding, and the quality of embedding is improved. Matrix decomposition based methods use some matrices to represent the network and its attributes, and then decompose them to obtain network embedding. Grasep [8] obtains the k-order relationship information between vertices through matrix operation, and integrates this information into the new loss function, which is modified from the loss functions in Deepwalk and Node2vec, to obtain the network embedding. HOPE [9] obtains the high-order proximity matrix S in the network through matrix polynomial operation; then approximates S through methods such as PageRank; and finally obtains the network embedding by minimizing the loss function through singular value decomposition. TADW [10] proposes text-related DeepWalk by proving that DeepWalk is in fact equivalent to matrix factorization. MMDW [11] follows the method in TADW, writes DeepWalk in the form of matrix decomposition and then adds the regular term related to the max-margin classifier into the loss function, so as to achieve a better classification effect. HSCA [12] integrates information from network homogeneity, topology and node content to obtain effective network embedding. In general, deep learning-based approaches achieve the goal of learning network embedding by stacking multiple neural network layers to optimize their loss functions. Unlike LINE [6], which optimizes first-order and second-order proximities respectively, SDNE [13] uses an autoencoder structure to optimize first-order and second-order proximities simultaneously. SNE [14] uses two deep neural network models to process structure information and attribute information, and then outputs the processing results to the same hidden layers for network embedding learning.

The structure of a graph (network) is generally very irregular and can be considered as infinite dimensional data, so it does not have translational invariance. The surrounding structure of each node may be unique, and the data of such structure will make the traditional CNN ineffective. Therefore, the graph convolution network (GCN) [15], a variant of CNN, is proposed as a feature extractor on graph structure data to learn graph embedding. It is worth noting that as the depth of the neural network model increases, the effect of the GCN model decreases, which is due to the problem of excessive smoothing. Both GCN and GAT [16] aggregate the features of neighborhood nodes to obtain the embedding of target node. However, unlike GCN, which gives importance to nodes in the neighborhood of target node through the Laplacian matrix, GAT gives different importance to theses nodes using attention coefficients. Therefore, GAT can better integrate network structure information and feature information.

The above network embedding methods are carried out in Euclidean space, and they do not consider the fact that graph-structured data are non-Euclidean. Our approach considers the network as a discrete manifold, and uses the mathematical tools of Riemannian geometry to process the graph data. The contributions of this paper are: (i) We investigated the aggregation pattern for nodes’ information in the discrete manifold as opposed to Euclidean space. We found a geometrical quantity, Ricci curvature, which is better adapted to the polymerization process on the discrete manifolds. (ii) We propose a concrete implementation of the aggregation pattern mentioned above, the Ricci curvature-based graph convolutional neural network (RCGCN), for network embedding. Our model, namely, RCGCN, uses Ricci curvature to assign different importance to the nodes within the neighborhood of the target node. (iii) We conducted extensive experiments to compare RCGCN with baseline methods on several benchmarks.

## 2. Background

In this section, we review the related notion of curvature on the manifold. Then we introduce Ollivier’s coarse Ricci curvature, which generalizes Ricci curvature on the manifold to metric space by Wasserstein distance.

### 2.1. Sectional Curvature and Ricci Curvature

In differential geometry, the Riemannian curvature tensor or Riemannian tensor is the standard way of expressing the curvature of a Riemannian manifold. The curvature tensor is given by the following equation by Levi–Civita connection [17]:

(1) $$R_{m}\left(u,v\right)z=\nabla_{u}\nabla_{v}z-\nabla_{v}\nabla_{u}z-\nabla_{\left[u,v\right]}z,$$

where *u* and *v* are vector fields. As we can see in Equation (2), we can transform the Riemannian curvature tensor to a (0,4)-tensor with the help of the Riemannian metric *g*.

(2) $$R_{m}\left(u,v,z,w\right)=g\left(R_{m}\left(u,v\right)z,w\right).$$

Suppose that (M, g) is a n-dimensional Riemannnian manifold and $T_{p}M$ is a tangent space of *M* at a point *p*. For any two linearly independent vectors u,v, the quantity

(3) $$K\left(u,v\right)=-\frac{R(u,v,u,v)}{g\left(u,u\right)g\left(v,v\right)-g{\left(u,v\right)}^{2}}$$

is called Riemannian section curvature along section [u,v] at point *p*. Sectional curvature is the extension of Gaussian curvature in Riemannian geometry. Section curvature is an important intrinsic geometric quantity in Riemannian geometry, which reflects the degree of space bending. The Riemannian manifold of constant section curvature can be divided into three cases: hyperbolic space, Euclidean space and spherical space.

If *u* and *v* are two vectors in the tangent space $T_{p}M$, the so-called Ricci curvature tensor Ric(u,v) defines a linear map $u_{p}\to R\left(u_{p},v\right)u$ in the tangent space $T_{p}M$. Specifically, Ric(X,Y) is the trace of the Riemannian curvature tensor [17].

(4) $$Ric\left(u,v\right)=trace(z\to R_{m}\left(z,v\right)u).$$

For any non-zero tangent vector *u*, the quantity

(5) $$Ric\left(u\right)=\frac{Ric(u,u)}{g(u,u)}$$

is the Ricci curvature at point *p* along the tangent vector *u*. Obviously, Ricci curvature is the average of the sectional curvature of the plane containing *u*.

### 2.2. Coarse Ricci Curvature

**Figure 1:**
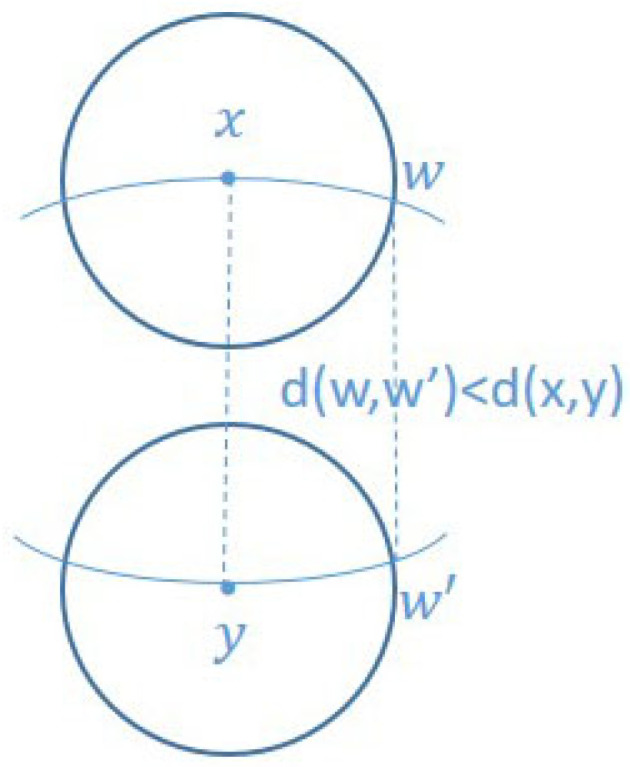
Illustration of the notion of curvature.

In Riemannian geometry, the Ricci curvature determines the volume of the overlap between two small balls. If the Ricci curvature is positive, it means that the balls are closer than their centers in terms of the transportation distance [18,19], as shown in Figure 1.

Let x,y be two points which are closely located in a Riemannian manifold, then (xy) is a tangent vector. Let *w* is a tangent vector at *x*, and we can get the tangent vector $w^{\prime }$ by parallel transport *w* from *x* to *y*. Following the geodesics emanating from x,w and $y,w^{\prime }$, the geodesics will get closer if curvature is positive, and they will move away in the case of negative curvature. Ricci curvature on (xy) is the average of the curvature aforementioned in all directions *w* at *x*. We can imagine that there are two small spheres $S_{x}$ and $S_{y}$ centered at the points x,y respectively. If the Ricci curvature of (*xy*) is positive, the distance between the centers of two small spheres is less than d(x,y). It is important to note that this series of concepts comes from the continuous scene.

**Figure 2:**
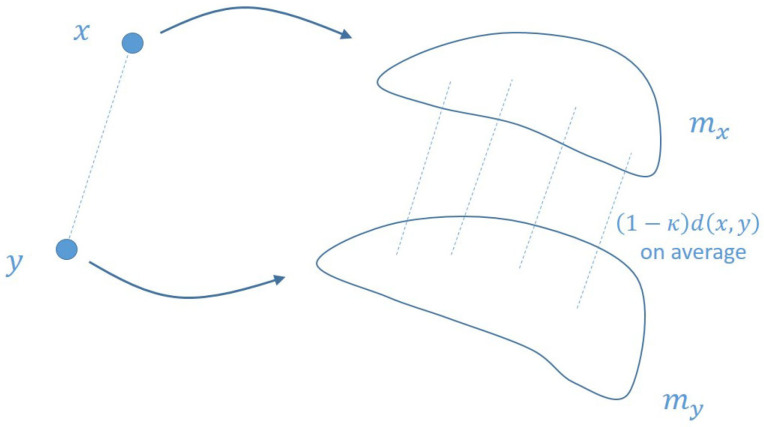
Illustration of the generalized concept of curvature.

To analyze the more general situation, Y. Ollivier [19] replaces the sphere $S_{x}$ centered at *x* by a probability measure $m_{x}$ depending on *x*, as is shown in Figure 2.

**Definition** **1** ([19,20])**.** *Let (X,d) be a metric space and let μ and ν be two probability measures on X. The $L^{1}$ Wasserstein distance between μ and ν is*

(6) $$W_{1}\left(\mu ,\nu \right)=\underset{\pi \in \prod (\mu ,\nu )}{\inf}\int_{X\times X}d\left(x,y\right)d\pi \left(x,y\right).$$

*where ∏(μ,ν) is the set of measures on X×X projecting to μ and ν.*

**Definition** **2** ([19,20,21])**.** *Let (X,d) be a metric space equipped with a probability $m_{x}$ for each x∈X. Let x,y be two points in X. The coarse Ricci curvature of (xy) is*

(7) $$\kappa \left(x,y\right)=1-\frac{W_{1}\left(m_{x},m_{y}\right)}{d(x,y)}.$$

The generalized concepts can be applied to discrete scenarios, such as networks. [22] also uses this notion of Ricci curvature. We now consider a network (or graph) *G* as a metric space, where *d* is the natural distance in graph.

Next we give two definitions of the scalar curvature of vertices. In the following, we will adopt the latter definition of scalar curvature of vertices.

**Definition** **3** ([23])**.** *Let G=(V,E) be a network and x∈V is a node in the network. Let Γ(x) be the set of neighbors of *x*. The scalar curvature of node *x* is*

(8) $$S_{R}\left(x\right)=\frac{1}{d_{x}}\sum_{y\in \Gamma (x)}\kappa \left(x,y\right).$$

*where $d_{x}$ is the degree of node *x*.*

**Definition** **4** ([24]). *Let G=(V,E) be a network and x∈V is a node in the network. Let Γ(x) be the set of neighbors of *x*. The scalar curvature of node *x* is*

(9) $$S_{R}\left(x\right)=\sum_{y\in \Gamma (x)}\kappa \left(x,y\right).$$

## 3. Methods

### 3.1. Problem Setting

Let G=(V,E,X,T) be an attributed network with labels, where *V* denotes the set of nodes, *E* represents the set of edges, *X* denotes the set of attributes associated with nodes and *T* represents the set of labels associated with nodes.

**Definition** **5.** *Given a network G=(V,E,X,T), we aim to define a mapping function $f:v_{i}\to y_{i}\in R^{d}$ for every node $v_{i}\in V$, where d<<|V| and f preserve the similarity of network topology, node attributes and node labels.*

### 3.2. Neighborhood Aggregation on the Manifold by RCGCN

Inspired by the GCN and Ricci curvature, we propose the novel method RCGCN.

**Figure 3:**
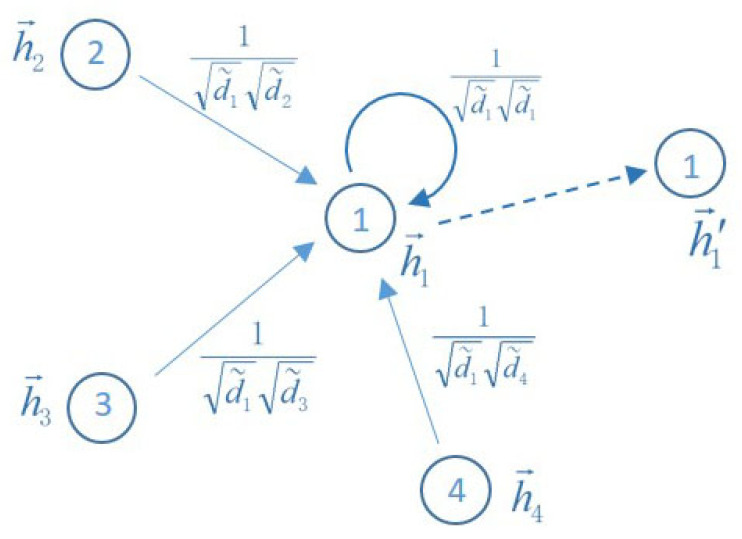
Diagram of the aggregation mode.

Let us first review the layer-wise propagation rule of the graph convolutional network, which is given as follows:

(10) $$H^{\left(l+1\right)}=g\left({\tilde{D}}^{-\frac{1}{2}}\tilde{A}{\tilde{D}}^{-\frac{1}{2}}H^{\left(l\right)}W^{\left(l\right)}\right).$$

where $\tilde{A}=A+I_{N}$ is the adjacency matrix of network *G* with self-connections, $I_{N}$ is the identity matrix, ${\tilde{D}}_{ii}=\sum_{j}{\tilde{A}}_{ij}$, $W^{\left(l\right)}$ is a trainable weight matrix, g(·) represents an activation function and $H^{\left(l\right)}$ is the matrix of activations in the *l*th layer with $H^{\left(0\right)}=X$. If we convert the matrix form to the component form, we get the following equation:

(11) $$h_{i}^{\left(l+1\right)}=g\left(\sum_{j\in N_{i}}\frac{1}{\sqrt{\tilde{d_{i}}\tilde{d_{j}}}}h_{j}^{\left(l\right)}W^{\left(l\right)}+\frac{1}{\sqrt{\tilde{d_{i}}\tilde{d_{i}}}}h_{i}^{\left(l\right)}W^{\left(l\right)}\right),$$

as is shown in Figure 3.

**Definition** **6** ([22])**.** *For any α∈[0,1] and any node x, the probability measure $m_{x}^{\alpha }$ is defined as follows:*

(12) $$m_{x}^{\alpha }\left(u\right)=\left\{\begin{array}{cc} \alpha , & if\;u=x\\ \frac{\left(1-\alpha \right)}{d_{x}}, & if\;u\in \Gamma (x)\\ 0, & otherwise, \end{array}\right.$$

*where Γ(x) represents the set of neighbors of x, and $d_{x}$ is the degree of node *x*.*

**Remark** **1.** *(i) By combining Definitions 2 and 6, we can calculate the curvature of the edges in the network. (ii) The above probability measures can be viewed as distributing the unit mass onto the unit sphere, and they are the discrete analogs of the Riemannian geometric continuous objects in Figure 1 and Figure 2. We will elaborate on these two points in the following paragraphs.*

According to Definition 2, we can see that the Ricci curvature is related to the optimal transport distance, namely, Wasserstein distance, which in turn is related to the transport plan between two probability measures in some metric space. The research object of this paper is a graph (network), so the metric space *X* in Definitions 1 and 2 is graph G=(V,E), and d(x,y) is the usual graph distance, that is, the number of edges on the shortest path connecting *x* and *y* in the graph. Let us consider any edge (x,y) in the network, which is associated with nodes *x* and *y* respectively. Intuitively, we can imagine that there are two discrete unit spheres at *x* and *y*, namely, $S_{x}$ and $S_{y}$. It is worth noting that the discrete unit spheres mentioned here are the discrete versions of the unit spheres in Figure 1. For the sake of illustration, we will analyze only the discrete unit sphere $S_{x}$.

First of all, we should notice that Formula (12) indicates the mass distribution on the discrete unit spheres $S_{x}$, and the total mass of $S_{x}$ is 1. The mass of α is distributed in the center of the sphere, i.e., node *x*; the remaining mass of 1−α is uniformly distributed on the surface of the sphere $S_{x}$, i.e., the direct neighbors of node *x*; the mass placed in other nodes of the network is 0. If α is equal to 0, then the mass at the center of the sphere is zero, and the rest of the mass is distributed on the spherical surface. If α is equal to 1, then the mass is completely concentrated at the center of the sphere $S_{x}$, namely, node *x*. Secondly, let us consider the optimal transfer issue between $S_{x}$ and $S_{y}$. Initially, the mass is distributed in terms of $m_{x}^{\alpha }\left(u\right)$; that is, the mass at node *x* is α, and the mass at each neighbor of *x* is $\frac{\left(1-\alpha \right)}{d_{x}}$. At this point, there is no mass distributed at the unit sphere $S_{y}$. We need to make a transportation plan to move the mass from $S_{x}$ to $S_{y}$, and the final mass distribution is consistent with $m_{y}^{\alpha }\left(u\right)$. Obviously, there may be many transport schemes that meet the above requirements. However, what we want to find is the transmission plan with the least cost, and the least cost corresponding to the optimal scheme is the optimal transport distance, namely, Wasserstein distance. Based on the above analysis, we can give the following discrete version of Wasserstein distance in Definition 1 as follows:

(13) $$\begin{array}{cc} W_{1}\left(m_{x}^{\alpha },m_{y}^{\alpha }\right) & =\underset{x^{\prime }\in N\left(x\right),y^{\prime }\in N\left(y\right)}{\inf}\pi \left(x^{\prime },y^{\prime }\right)d\left(x^{\prime },y^{\prime }\right)=\min_{x^{\prime }\in N\left(x\right),y^{\prime }\in N\left(y\right)}\pi \left(x^{\prime },y^{\prime }\right)d\left(x^{\prime },y^{\prime }\right)\\ s.t. & \sum_{y^{\prime }\in N\left(y\right)}\pi \left(x^{\prime },y^{\prime }\right)=m_{x}^{\alpha }\left(x^{\prime }\right),\;\sum_{x^{\prime }\in N\left(x\right)}\pi \left(x^{\prime },y^{\prime }\right)=m_{y}^{\alpha }\left(y^{\prime }\right) \end{array}$$

where N(x) is the union of *x* and Γ(x), and N(y) is the union of *y* and Γ(y).

**Figure 4:**
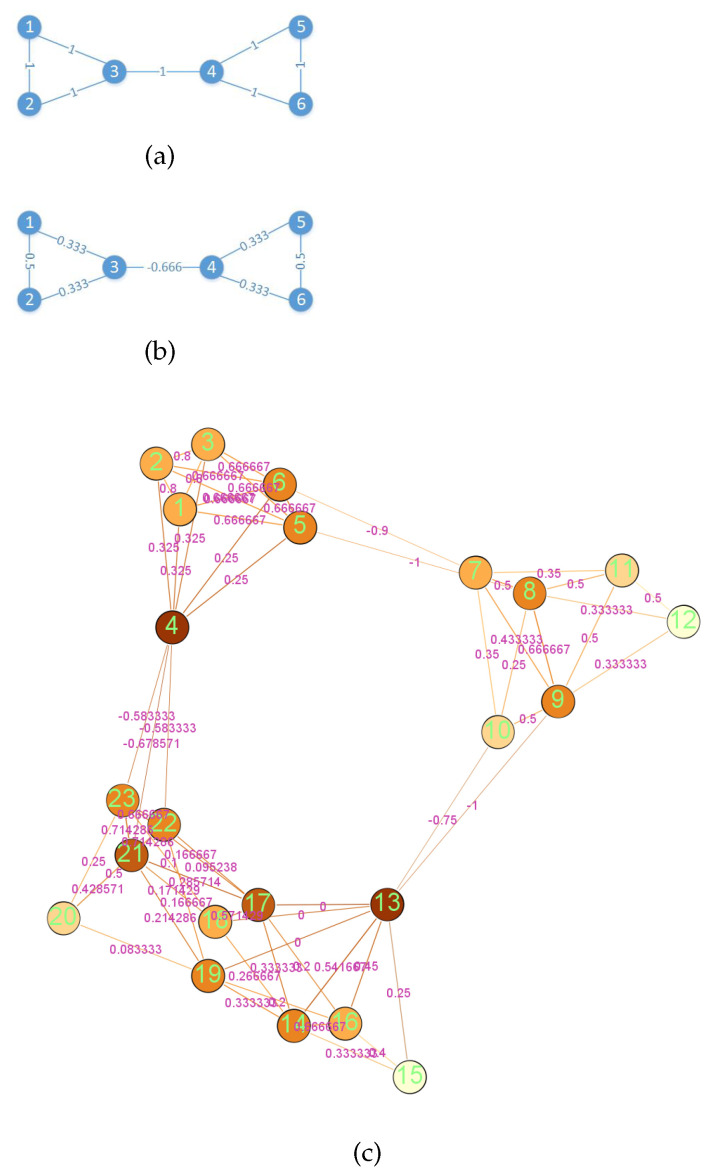
Illustration of coarse Ricci curvature and manifold.

We note that Equation (13) is a linear programming problem and that the nodes involved in the calculation are limited to those nodes in the neighborhood of x,y, which makes the Wassertein distance and thus the Ricci curvature of the edge (x,y) easy to calculate. Next, let us observe the properties of Ricci curvature. We compare the two network diagrams in Figure 4. Figure 4a shows an unweighted and undirected network with six nodes, whose weights of the edges are all 1. Figure 4b is a curvature diagram of the network in Figure 4a, where the weight of each edge is its Ricci curvature. According to the calculation method in Figure 3, we can derive the representation of node 3 is as follows:

(14) $${\vec{h}}_{3}=\frac{1}{\sqrt{3}\sqrt{4}}{\vec{h}}_{1}+\frac{1}{\sqrt{3}\sqrt{4}}{\vec{h}}_{2}+\frac{1}{\sqrt{4}\sqrt{4}}{\vec{h}}_{4}$$

According to Equation (14), the influences of nodes 1, 2 and 4 on the representation of node 3 are roughly the same. However, when we consider the problem mentioned above and add the additional information of the community, we can see that the representation in Equation (14) is not accurate enough. For example, there are two communities in the network of Figure 4a. Obviously, one community consists of nodes 1, 2 and 3, and the other community is composed of nodes 4, 5 and 6. Compared with the nodes of different communities, the nodes of the same community are more closely related to each other, and their embedded representations should be closer together. Consequently, the embedded representations of nodes 1, 2 and 3 should be close to each other, while the representations of node 3 and node 4 should be mutually far away. Additionally, Equation (14) does not reflect this fact. Therefore, we want to find a quantity that reflects this fact by giving different importance to the nodes in the neighborhood of the target node. The importance of nodes described here can be reflected in the weighting coefficients of Equation (14). First, we give the curvature network corresponding to the network in Figure 4a, that is, replace the weights of the edges in Figure 4a with the corresponding curvature, as shown in Figure 4b. In addition, to facilitate observation, we also present a larger artificial network than the network in Figure 4a, i.e., Figure 4c. Figure 4c is a curvature diagram of a network with 23 nodes, where each edge’s weight is its Ricci curvature. It is clear that the network in Figure 4c can be roughly divided into three communities. The weights of edges in Figure 4c are the curvature values of its original network edges. From Figure 4b,c, we observe the following results: (i) Edges within a community have positive coarse Ricci curvature. (ii) Although the Ricci curvature values of edges within the same community are all positive, they are not constant. (iii) Edges between communities have negative coarse Ricci curvature. Next, let us analyze the reasons for this phenomenon, and how to use this phenomenon to help us assign different importance values to the nodes in the neighborhood of the target node, so that the RCGCN model can better aggregate the features of the neighborhood nodes.

Let us consider an edge (x,y) in the network, which is associated with nodes x and y. If x and y are in the same community, then usually their neighborhoods will overlap—that is, there will be common neighbors. Hence, when the discrete sphere with the center of x transfers the mass to the discrete sphere with the center of y through the optimal transport plan, there will be more shortcuts to take. This will make the optimal cost in Equation (13), that is, the Wassertein distance, less than the length of the edge (x,y). As a consequence, the Ricci curvature of the edge (x,y) is positive. Moreover, the larger the overlap of the neighborhoods of x and y, the greater the curvature value of edge (x,y). On the other hand, if x and y are in different communities, that is, edge (x,y) is a bridge between the two communities, then according to Equation (13), in order for the mass placed at x’s neighbor x′ to be transferred to y’s neighbor y′, the path taken must include bridge (x,y). In this way, the Wasserstein distance must exceed d(x,y), so the curvature of the corresponding edge (x,y) will be less than 0. Subsequently, coarse Ricci curvature can reflect the similarity between nodes; i.e., the larger the curvature value of the edge is, the more similar the nodes connected by the edge are. In other words, the embedded representations of nodes connected by a edge with large curvature value should be close to each other; vice versa. Thus, we intend to replace the coefficients in Equation (14), which reflects the aggregation pattern, with the curvature of the corresponding edges. However, it can be seen from Figure 4b,c that the curvature values of some edges in the network will be negative, which is not convenient for subsequent data processing. Therefore, we considered replacing the coefficients in Equation (14) with the values of the curvature of the edges after some transformation. Thereupon, we chose Equation (16) to perform this transformation. Additionally, we note that the function in Equation (16) is monotonically increasing, so that the relationship between the magnitude of curvature values remains unchanged.

### 3.3. RCGCN Architecture

**Figure 5:**
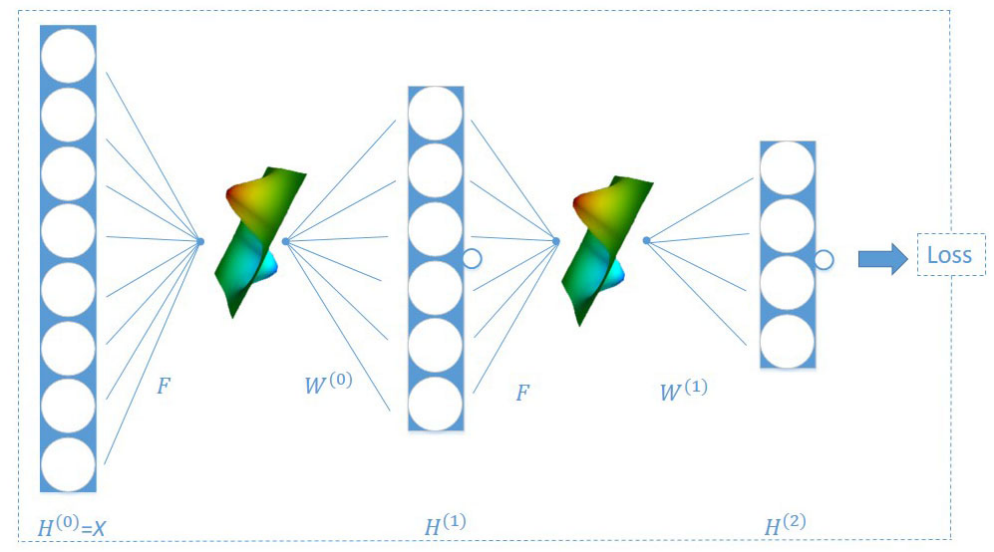
Ricci curvature-based graph convolutional neural network (RCGCN) architecture.

As shown in Figure 5, RCGCN, like GCN [15], adopts a two-layer neural network architecture. Layer 0 of RCGCN is the input layer, and the input data are the features of network nodes, namely, $H^{\left(0\right)}=X$. In this model, the input features *X* are aggregated by the corresponding normalized matrix of the matrix in Equation (18), and the representation of the first layer $H^{\left(1\right)}$ is obtained. Layer 2 of the model takes the representation $H^{\left(1\right)}$ of the previous layer as its input, aggregates it again and then obtains the representation of layer 2. Finally, the representation $H^{\left(2\right)}$ of the second layer is used to construct the loss function, and the network embedding is obtained by training. Next, we give the details of the RCGCN model.

We will make a preliminary modification to Equation (10). Firstly, define a sigmoid function as follows:

(15) $$sig\left(x\right)=\frac{1}{1+e^{-x}}.$$

Then, let $\bar{C}=\left[{\bar{c}}_{ij}\right]$ be the matrix whose elements ${\bar{c}}_{ij}$ are coarse Ricci curvature values of edges in the network and $A=[a_{ij}]$ be the adjacent matrix of the network. For the convenience of data processing, we convert the curvature matrix $\bar{C}$ to the matrix $C=[c_{ij}]$, whose elements are defined as follows:

(16) $$c_{ij}=\left\{\begin{array}{cc} \lambda \cdot sig({\bar{c}}_{ij}), & if\;a_{ij}=1\\ 0, & if\;a_{ij}=0, \end{array}\right.$$

where λ is a scale factor and is set to 1.8 in the experiments of this paper.

**Remark** **2.** *For the convenience of subsequent expression, combining with Equation (16), we modify the concept of scalar curvature of node v in Definition 4, and call $\sum_{u\in \Gamma (v)}c_{uv}$ the scalar curvature of node v and denote it as $S_{R}^{+}\left(v\right)$.*

In order to aggregate the features of the nodes in the neighborhood of the target nodes, we combine the curvature matrix *C* with the adjacency matrix *A*, which represents the original topology of the network. Note that the elements in the curvature matrix *C* come from Equation (16). In addition, we also consider the influence of the scalar curvature of nodes on the final representation. *S* in Equation (16) is a diagonal matrix whose diagonal elements are the values of the scalar curvature mapped by the sigmoid function, i.e., $S=diag(Sig\left(S_{R}^{+}\left(v_{1}\right)\right),Sig\left(S_{R}^{+}\left(v_{2}\right)\right),\cdots ,Sig\left(S_{R}^{+}\left(v_{n}\right)\right))$, where $v_{1},v_{2},\ldots ,v_{n}$ are the nodes in the network. Then let us define the transmission matrix *F* as follows:

(17) $$F=\beta_{0}\cdot A+\beta_{1}\cdot C+\omega \cdot S,$$

Then we normalize the matrix *F*, and we also denote it as *F* after the normalization. We give the following layer-wise propagation rule of RCGCN:

(18) $$H^{\left(l+1\right)}=g\left(FH^{\left(l\right)}W^{\left(l\right)}\right).$$

On the other hand, we will construct the new loss function of our neural network model. Let us first review the loss function for GCN, which is the cross-entropy error and defined as follows:

(19) $$L_{1}=-\sum_{l\in y_{L}}\sum_{f=1}^{M}Y_{lf}lnZ_{lf},$$

where *M* is the number of labels, $y_{L}$ is the set of node indices that own labels, *Y* is the vector representation of labels and *Z* is the output of GCN. Now we consider the following equations:

(20) $$Reg_{1}=\sum_{i,j}c_{ij}{∥f\left(X_{i}\right)-f\left(X_{j}\right)∥}^{2},$$

where $c_{ij}$ comes from Equation (16), and it is the value of the curvature of the edge mapped by the function in Equation (16), and $X_{i}$ and $X_{j}$ are node feature vectors. Another constraint is

(21) $$Reg_{2}={∥f{\left(X\right)}^{T}Df\left(X\right)-I∥}^{2}.$$

where *D* is a diagonal matrix whose diagonal elements are the values of the scalar curvature mapped by the sigmoid function, i.e., $D=diag(Sig\left(S_{R}\left(v_{1}\right)\right),Sig\left(S_{R}\left(v_{2}\right)\right),\cdots ,Sig\left(S_{R}\left(v_{n}\right)\right))$, where $v_{1},v_{2},\ldots ,v_{n}$ are the nodes in the network, and *X* is a feature matrix.

Consequently, the loss function of our neural network model RCGCN is defined as follows:

(22) $$L=L_{1}+\gamma_{1}\cdot Reg_{1}+\gamma_{2}\cdot Reg_{2}.$$

In the training process of the model, we use the gradient method to continuously reduce the loss function L mentioned above. From Equation (20), we can make the following observations: if $c_{ij}$ is small, then the difference between the representations of nodes $v_{i},v_{j}$ can be large during the process of optimization, and if $c_{ij}$ is relatively large, the representations of nodes $v_{i},v_{j}$ should be as close as possible. Moreover, we must add the regular term $Reg_{2}$ to the loss function, which can give a soft constraint to the node representations in Equation (22).

## 4. Experiment

We tested our neural network model in a number of experiments: semi-supervised node classification in the common datasets.

### 4.1. Datasets

We ran our model on five graph datasets: Citeseer, Cora, Cornell, Texas and Wisconsin. Dataset statistics are listed in Table 1. The column named Nodes in Table 1 has the numbers of nodes in the networks, while the column named Edges has the numbers of edges in the networks. The column named Classes in Table 1 shows the number of classes that the nodes in the network belong to. The column named Features corresponds to the dimensions of the node’s Feature.

Citeseer and Cora are citation network datasets. In these citation networks, each node represents a paper and each edge denotes a citation between two papers. Each node is assigned a vector composed of word frequency as its feature vector, while the label on each node indicates the classification to which the corresponding paper belongs. The papers in Cora dataset are classified into five categories, namely, neural networks, rule learning, reinforcement learning, probabilistic methods, theory, genetic algorithms and case based, while the papers in citeseer dataset are divided into five types, which are agents, IR, DB, AI, ML and HCI.

Cornell, Texas and Wisconsin are the subdatasets of WebKB, which is a webpage dataset collected by Carnegie Mellon University. These three subdatasets constitute the attribute networks in the following way: The nodes are web pages in the subdatasets, and the edges represent hyperlinks between them. The node feature is the word bag representation of the corresponding web page, and the node label corresponds to the category of the web page. The nodes in these networks are divided into five categories: faculty, course, student, staff and project.

### 4.2. Setup of Baseline Methods

Our experiment evaluated RCGCN using a standard semi-supervised learning task: label classification on nodes. For this task, we evaluated the performance of RCGCN against the following baselines.

*Deepwalk* [4]: It trains the representation of each node in the network using the skip-gram model, treating sequences of nodes from truncated random walks as sentences and nodes as words.

*Planetoid* [25]: It trains the samples to predict both the category labels of the samples and the contexts in the graph. It learns node embedding by optimizing for class label loss and context loss.

*GCN* [15]: It is a convolutional neural network using a local first-order approximation of spectral convolution. It aggregates the features of neighborhood nodes into those of target nodes by means of convolution.

*GAT* [16]: It is a variant of GCN. In the process of feature aggregation of neighborhood nodes, GAT uses the attention coefficient to assign importance to neighborhood nodes, which is different from what GCN does—it uses a Laplace matrix to achieve this.

### 4.3. Semi-Supervised Node Classification

Similarly to the evaluation methods in GCN, we evaluated the performance of RCGCN by comparing the node classification performances of RCGCN and the baselines on the aforementioned datasets.

The experimental results are listed in Table 2. The numbers in Table 2 represent the percentages of node classification accuracy. The numbers in Table 2 that represent the best performance are shown in bold. The methods listed in Table 2 are RCGCN and baseline methods: Deepwalk [4], Planetoid [25], GCN [15] and GAT [16]. The hidden layer dimensions of Deepwalk, Planetoid, GCN and RCGCN were all set to 16. Following the experimental setup of the authors, GAT used eight attention heads, and each attention head had an 8-dimensional hidden layer representation. Thus, the hidden layer dimensions of GAT are essentially 64. Meanwhile, it is noted that GCN, GAT and RCGCN all adopted a two-layer neural network architecture. For Cora, the super parameters $\gamma_{1},\gamma_{2}$ in Equation (22) and the super parameters $\beta_{0},\beta_{1}$ and ω in Equation (17) were set as $2\times 10^{-6},3\times 10^{-7},0.13,0.01$ and 0.13 respectively. For Citeseer, the super parameters mentioned above were $2\times 10^{-4},1.5\times 10^{-5},0.175,0.02$ and 1 respectively. For Cornell, they were $2\times 10^{-3},3\times 10^{-3},0.01,0.015$ and 1 respectively. For Texas, they were $2\times 10^{-3},3\times 10^{-3},0.005,0.44$ and 1 respectively. For Wisconsin, they were $2\times 10^{-3},3\times 10^{-3},0.005,0.04$ and 1 respectively.

**Figure 6:**
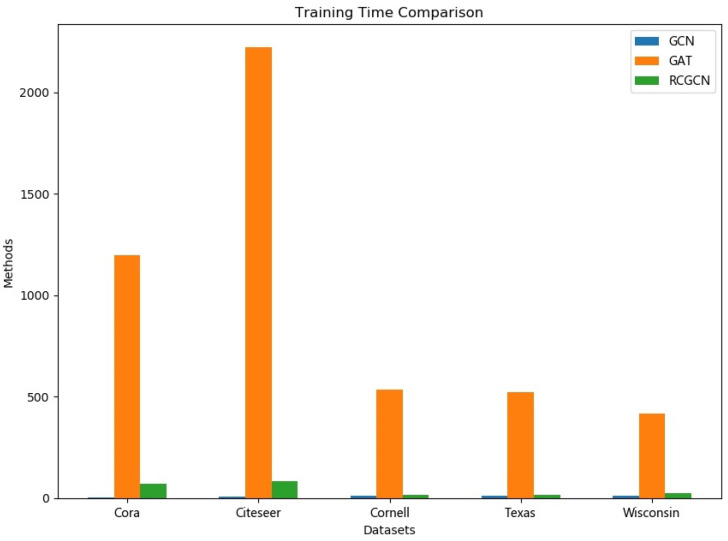
Training time comparison.

Our results show that RCGCN achieved the best performance on all five datasets, except that the classification accuracy on the CORA dataset was slightly behind that of GAT. However, it can be seen from Figure 6 that the training time required by GAT is much longer than that of RCGCN. We can see that the RCGCN in the last row of the Table 2 has a significant performance gain compared to the other methods. For datasets Cora and Citeseer, the performance gains of RCGCN relative to GCN reached 0.6% and 4.1% respectively, while for datasets Cornell, Texas and Wisconsin, the gains were 26.8%, 34.7% and 61.5%.

### 4.4. Training Time Comparison

RCGCN is an improved model based on GCN. Both RCGCN and GAT are variants of GCN. Therefore, we conducted further experiments to compare the training times of GCN, GAT and RCGCN. The experiments here were conducted on a CPU, using a laptop with an Intel Core i7 2.8GHz processor and 8 G RAM. Figure 6 summarizes the results. It can be seen from Figure 6 that the training times of GCN and RCGCN in the datasets Cornell, Texas and Wisconsin were about the same. The training time of RCGCN was slightly longer than that of GCN for the datasets Cora and Citeseer, mainly because RCGCN needs to calculate curvature in the first epoch of training. On all five datasets, GAT was trained for a much longer time than GCN and RCGCN.

## 5. Conclusions and Future Work

We introduced RCGCN, a novel architecture that combines the neural network and Riemannian geometric methods. We consider the network as a discrete manifold, making use of the information of probability measure manifold based on the original manifold. By expanding the neighborhood of target node, we obtain high-order information about the target node. Based on the above information, we train the novel model using a new loss function, which can help to get a better representation of the nodes. Experiments on several benchmark datasets showed that the RCGCN model can effectively encode network structure and node characteristics for semi-supervised classification. In the future, we can consider improving the model in the following aspects: (i) We will further excavate the geometric information of the underlying manifold to improve the aggregation of network information. (ii) We will consider adding long-range information of the network to improve the model. (iii) We will try to extend the model for graph classification rather than node classification.

## Figures and Tables

**Table 1:** Dataset statics.

Dataset	Type	Nodes	Edges	Classes	Features
Cora	Citation network	2708	5429	7	1433
Citeseer	Citation network	3327	4732	6	3703
Cornell	WebKB	183	295	5	1703
Texas	WebKB	183	309	5	1703
Wisconsin	WebKB	251	499	5	1703

**Table 2:** Summary of results in the form of classification accuracy (percent).

Method	Cora	Citeseer	Cornell	Texas	Wisconsin
DeepWalk [4]	67.2	43.2	58.38	57.84	50.39
Planetoid [25]	75.7	64.7	45.41	63.24	63.14
GCN [15]	81.5	70.3	62.43	60.81	51.37
GAT [16]	**82.4**	72.6	68.92	62.16	38.43
RCGCN	82.0	**73.2**	**79.19**	**81.89**	**82.94**

## Data Availability

Data sharing not applicable.
